# Determining the Specificity of Cascade Binding, Interference, and Primed Adaptation *In Vivo* in the *Escherichia coli* Type I-E CRISPR-Cas System

**DOI:** 10.1128/mBio.02100-17

**Published:** 2018-04-17

**Authors:** Lauren A. Cooper, Anne M. Stringer, Joseph T. Wade

**Affiliations:** a Department of Biomedical Sciences, School of Public Health, University at Albany, Albany, New York, USA; b Wadsworth Center, New York State Department of Health, Albany, New York, USA; Max Planck Institute for Infection Biology

**Keywords:** CRISPR, Cas3, Cascade, PAM, priming, protospacer

## Abstract

In clustered regularly interspaced short palindromic repeat (CRISPR)-Cas (CRISPR-associated) immunity systems, short CRISPR RNAs (crRNAs) are bound by Cas proteins, and these complexes target invading nucleic acid molecules for degradation in a process known as interference. In type I CRISPR-Cas systems, the Cas protein complex that binds DNA is known as Cascade. Association of Cascade with target DNA can also lead to acquisition of new immunity elements in a process known as primed adaptation. Here, we assess the specificity determinants for Cascade-DNA interaction, interference, and primed adaptation *in vivo*, for the type I-E system of Escherichia coli. Remarkably, as few as 5 bp of crRNA-DNA are sufficient for association of Cascade with a DNA target. Consequently, a single crRNA promotes Cascade association with numerous off-target sites, and the endogenous E. coli crRNAs direct Cascade binding to >100 chromosomal sites. In contrast to the low specificity of Cascade-DNA interactions, >18 bp are required for both interference and primed adaptation. Hence, Cascade binding to suboptimal, off-target sites is inert. Our data support a model in which the initial Cascade association with DNA targets requires only limited sequence complementarity at the crRNA 5′ end whereas recruitment and/or activation of the Cas3 nuclease, a prerequisite for interference and primed adaptation, requires extensive base pairing.

## IMPORTANCE

Many bacterial and archaeal species encode CRISPR-Cas immunity systems that protect against invasion by foreign DNA. In the Escherichia coli CRISPR-Cas system, a protein complex, Cascade, binds 61-nucleotide (nt) CRISPR RNAs (crRNAs). The Cascade complex is directed to invading DNA molecules through base pairing between the crRNA and target DNA. This leads to recruitment of the Cas3 nuclease, which destroys the invading DNA molecule and promotes acquisition of new immunity elements. We made the first *in vivo* measurements of Cascade binding to DNA targets. Thus, we show that Cascade binding to DNA is highly promiscuous; endogenous E. coli crRNAs can direct Cascade binding to >100 chromosomal locations. In contrast, we show that targeted degradation and acquisition of new immunity elements require highly specific association of Cascade with DNA, limiting CRISPR-Cas function to the appropriate targets.

## INTRODUCTION

Clustered regularly interspaced short palindromic repeat (CRISPR)-Cas (CRISPR-associated) systems are adaptive immune systems found in approximately 40% of bacteria and 90% of archaea (1). CRISPR-Cas systems are characterized by the presence of CRISPR arrays and Cas proteins. CRISPR arrays are genomic loci that consist of short repetitive sequences (“repeats”), interspaced with short sequences of viral or plasmid origin (“spacers”) (2–5). Spacers are acquired during a process known as “adaptation,” in which a complex of Cas1 and Cas2 integrates invading DNA into a CRISPR array, effectively immunizing the organism from future assault by the invader (6). In the archetypal type I-E CRISPR system of Escherichia coli, immunity occurs via two processes known as “biogenesis” and “interference.” During biogenesis, a CRISPR array is transcribed, and Cas6e processes the transcript into individual 61-nucleotide (nt) CRISPR RNAs (crRNAs) that each include a single 32-nt spacer sequence flanked by partial repeat sequences (7, 8). Individual crRNAs are then incorporated into Cascade, a protein complex composed of five different Cas proteins (Cse1 [Cas8e]-Cse2_2_-Cas7_6_-Cas5-Cas6e) (7, 9). During interference, Cascade complexes bind to target DNA sequences known as “protospacers” that are complementary to the crRNA spacer and are immediately adjacent to a short DNA sequence known as a “protospacer-associated motif” (PAM) (10) that is bound by Cse1 (11, 12). The crRNA bound by Cascade forms an R-loop with the target DNA, which in turn leads to recruitment of the Cas3 nuclease, DNA cleavage, and elimination of the invader (13–19).

For type I CRISPR-Cas systems, adaptation can occur by two mechanisms: “naive” and “primed.” Typically, naive adaptation requires only Cas1 and Cas2 (6, 20). Primed adaptation, in contrast, requires all of the Cas proteins and an existing crRNA (21). The molecular details of primed adaptation are poorly understood. Spacers acquired by primed adaptation correspond to locations on the same DNA molecule where the protospacer is located (21–24). Some type I CRISPR-Cas systems acquire spacers preferentially from one strand (21, 23, 25), whereas others acquire spacers from both strands (24, 26, 27). Primed adaptation has been proposed to involve translocation of Cas3 away from the Cascade-bound protospacer (21, 28).

There are conflicting reports on the relationship between interference and primed adaptation. Initially, it was proposed that primed adaptation occurs only when Cascade-protospacer interactions are suboptimal and cannot lead to interference, e.g., with a suboptimal PAM, or with mismatches in the PAM-proximal region of the protospacer known as the “seed” region (21, 23, 29–31). However, more-recent studies have shown that the presence of at least some protospacers can lead to both interference and primed adaptation, indicating that the requirements for interference and primed adaptation overlap (24, 32, 33).

Prior to interference or primed adaptation, Cascade must bind to the target protospacer. This requires an interaction between Cse1 and the PAM as well as base pairing between the crRNA and protospacer DNA (11, 12, 14). PAM recognition is required for both Cascade binding and later recruitment and activation of Cas3 (11, 13, 16, 19). Changes to the optimal PAM weaken Cascade binding to a protospacer (34). Nonetheless, some suboptimal PAMs are sufficient for interference, albeit they show lower efficiency than the optimal PAM (31). Sequences within the crRNA spacer are also required for initial binding of Cascade to a protospacer; mutations in positions 1 to 5 and positions 7 to 8 adjacent to the PAM of the protospacer (the seed sequence) reduce the affinity of Cascade for the protospacer (29).

Reports of the sequence determinants associated with Cascade binding, interference, and primed adaptation are conflicting (16, 18, 23, 31, 35). In particular, the impact of extensive mismatches in the crRNA:DNA hybrid on Cascade binding and primed adaptation is unclear (16, 35). Importantly, association of Cascade with protospacer DNA has not previously been studied in an *in vivo* context. Here, we used chromatin immunoprecipitation sequencing (ChIP-seq) to perform the first *in vivo* assessment of Cascade binding to its DNA targets. Our data show that base pairing between the crRNA and protospacer with as few as 5 nt in the seed region, coupled with an optimal PAM, is often sufficient for Cascade binding. Hence, crRNAs, including those transcribed from the native E. coli CRISPR loci, drive off-target binding at over 100 chromosomal sites. If Cascade binding to DNA were sufficient for interference or primed adaptation, these off-target binding events would likely be catastrophic for the bacterium (36, 37). However, we show that extensive base pairing between the crRNA and protospacer from the PAM-proximal end is required for efficient interference and primed adaptation. Thus, under native conditions, Cascade samples potential DNA target sites but limits nuclease activity to protospacers that meet a higher specificity threshold that would be expected only of on-target sites.

## RESULTS

### An AAG PAM and seed matches are sufficient for Cascade binding to DNA target sites *in vivo.*

**FIG 1:**
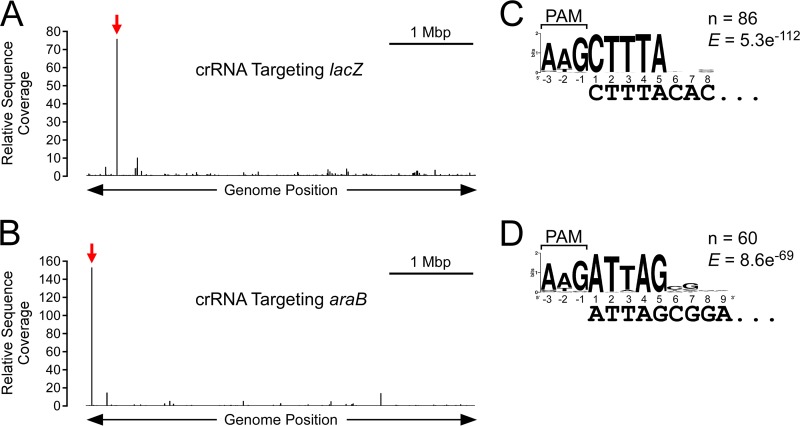
Extensive off-target Cascade binding in E. coli. (A) Binding profile of Cse1 across the E. coli genome, as determined by ChIP-seq, for Cse1-FLAG_3_ cells (AMD543) carrying a plasmid expressing crRNA targeting the *lacZ* promoter region (pCB380). The graph indicates the relative levels of sequence read coverage (see Materials and Methods for details) across the genome in a Cse1 ChIP-enriched sample. Bar, 1 Mbp. The location of the on-target binding site at the *lacZ* promoter is shown by the red arrow. (B) Binding profile of Cas5 across the E. coli genome, as determined by ChIP-seq, for FLAG_3_-Cas5 cells (AMD554) with a plasmid expressing crRNA targeting the *araB* promoter region (pCB381). The location of the on-target binding site at the *araC* promoter is shown by the red arrow. (C) Enriched sequence motif associated with off-target Cascade binding sites in cells targeting the *lacZ* promoter, as determined by MEME. The likely PAM sequence is indicated. The number of identified motifs and the MEME E value are shown. (D) Enriched sequence motif associated with off-target Cascade binding sites in cells targeting the *araB* promoter, as determined by MEME. The likely PAM sequence is indicated.

Previous studies of Cascade association with protospacer DNA have been performed *in vitro* using purified Cascade and crRNA. To determine the *in vivo* target specificity of E. coli Cascade, we used ChIP-seq to map the association of Cse1-FLAG_3_ and FLAG_3_-Cas5 (FLAG-tagged strains retain CRISPR function; see Fig. S1 in the supplemental material) across the E. coli chromosome in Δ*cas3* (interference-deficient) cells constitutively expressing all other *cas* genes and each of two crRNAs that target either the *lacZ* promoter or the *araB* promoter (both targets are chromosomal) (Fig. S2A and B). ChIP-seq data for Cse1 and Cas5 were highly correlated (*R*^2^ values of 0.93 to 0.99 for *lacZ*-targeting cells and 0.99 for *araB*-targeting cells), consistent with Cse1 and Cas5 binding DNA together in the context of Cascade. We detected association of Cascade with many genomic loci for each of the two spacers tested (Fig. 1A and B; see also Table S1 in the supplemental material). In all cases, the genomic region with strongest Cascade association was the on-target site at *lacZ* or *araB*. Off-target binding events occurred with <20% of the ChIP signal of on-target binding. To determine the sequence requirements for off-target Cascade binding with each of the two crRNAs used, we searched for enriched sequence motifs in the Cascade-bound regions, excluding the on-target site (Table S2). For both the *lacZ* and *araB* spacers, the most highly enriched sequence motif that we identified was a close match to the canonical PAM, AAG, on the nontarget strand, followed by 5 nt of sequence complementarity at the start of the crRNA seed region (Fig. 1C and D; cf. Fig. S2A and B). In some cases, we observed Cascade binding events associated with non-AAG PAMs; however, these sites were more weakly bound and/or had matches in seed region beyond position 5. For example, Cascade binding events targeted by the *araB* spacer were significantly more likely to have matches at positions 7 to 9 in cases where the PAM was not AAG (averages of 2.2 of 3 possible matches per target sequence in cases in which the PAM was not AAG [*n* = 19] and 1.2 of 3 possible matches in cases in which the PAM was AAG [*n* = 41]; Fisher's exact test *P* = 0.00005). We conclude that as few as 5 bp in the seed region, together with an AAG PAM, are sufficient for Cascade binding, with additional base pairing in or near the seed region increasing binding and/or overcoming the need for an AAG PAM.

10.1128/mBio.02100-17.1 FIG S1  FLAG_3_-tagged Cse1 and Cas5 are fully functional for primed adaptation. PCR amplification of the start of each of the CRISPR-I and CRISPR-II arrays was performed to detect primed adaptation in cells expressing *cas3* (pAMD191) and a plasmid-expressed crRNA that perfectly targets a sequence on the same plasmid (pAMD189). Adaptation was assessed for (i) MG1655 (no *cas* gene expression, except for plasmid-carried *cas3* [pAMD191]), (ii) MG1655 with constitutive expression of *cas* genes (AMD536), (iii) MG1655 *cse1*-FLAG_3_ with constitutive expression of *cas* genes (AMD543), and (iv) MG1655 FLAG_3_-Cas5 with constitutive expression of *cas* genes (AMD554). Download FIG S1, PDF file, 0.1 MB. Copyright © 2018 Cooper et al. 2018 Cooper et al. This content is distributed under the terms of the Creative Commons Attribution 4.0 International license.

10.1128/mBio.02100-17.2 FIG S2  crRNA spacers used in this study. (A) Sequence of the crRNA spacer targeting the *lacZ* promoter (pCB380). (B) Sequence of the crRNA spacer targeting the *araB* promoter (pCD381). (C) Sequence of the CRISPR-I array. Spacers 1, 3, 4, and 8 are underlined. (D) Sequence of the CRISPR-II array. Spacer 2 is underlined. (E) Sequence of a portion of the CRISPR-I spacer 8 crRNA-expressing plasmid (pLC008). Note that the sequence downstream of the second repeat (underlined) can be used as a spacer. Download FIG S2, PDF file, 0.1 MB. Copyright © 2018 Cooper et al. 2018 Cooper et al. This content is distributed under the terms of the Creative Commons Attribution 4.0 International license.

10.1128/mBio.02100-17.5 TABLE S1  Lists of ChIP-seq peak coordinates. Download TABLE S1, XLSX file, 0.03 MB. Copyright © 2018 Cooper et al. 2018 Cooper et al. This content is distributed under the terms of the Creative Commons Attribution 4.0 International license.

10.1128/mBio.02100-17.6 TABLE S2  Lists of regions used to search for enriched sequence motifs. Download TABLE S2, PDF file, 0.2 MB. Copyright © 2018 Cooper et al. 2018 Cooper et al. This content is distributed under the terms of the Creative Commons Attribution 4.0 International license.

### Extensive off-target Cascade binding driven by endogenous spacers.

**FIG 2:**
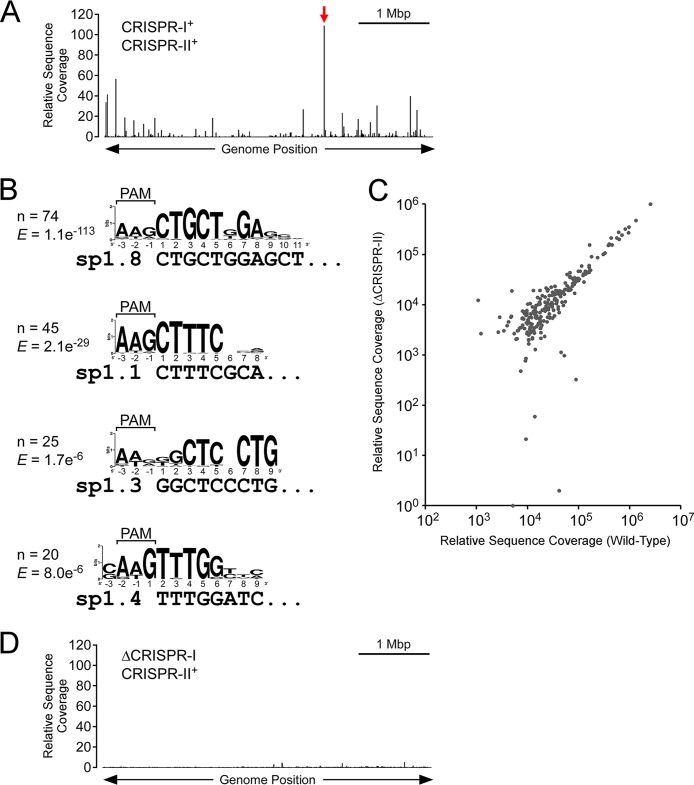
Endogenous crRNAs drive Cascade association with over 100 chromosomal sites. (A) Binding profile of Cse1 across the E. coli genome, as determined by ChIP-seq, for Cse1-FLAG_3_ cells (AMD543) expressing only endogenous crRNAs. The location of the binding site within *yggX* is shown by the red arrow. (B) Enriched sequence motifs associated with Cascade binding sites in cells expressing only endogenous crRNAs. The four motifs are associated with four of the CRISPR-I spacers, as indicated. The likely PAM sequence is also indicated. The number of identified motifs and the MEME E value are shown. (C) Comparison of Cascade binding events in Cse1-FLAG_3_ cells with both CRISPR arrays intact (AMD543) or with CRISPR-II deleted (LC060). Sequence read coverage is shown for CRISPR-1^+^ CRISPR-2^+^ cells (AMD543) and CRISPR-I^+^ ΔCRISPR-II cells (LC060), for all ChIP-seq peaks identified for either strain. (D) Binding profile of Cse1 across the E. coli genome, as determined by ChIP-seq, for Cse1-FLAG_3_ cells expressing only endogenous crRNAs but with CRISPR-I deleted (LC077).

We identified several sites of Cascade binding that were shared between cells targeting *lacZ* and cells targeting *araB*. These bound regions were not associated with sequences matching the seed regions of either crRNA. We reasoned that such off-target binding events may be due to Cascade association with the endogenous E. coli crRNAs. To test this hypothesis, we performed ChIP-seq analysis of Cse1-FLAG_3_, as described above, for cells expressing only the endogenous CRISPR RNAs from their native loci. Thus, we identified 188 binding sites for Cascade (Fig. 2A; see also Table S1). These sites were associated with four enriched sequence motifs, with each motif corresponding to a canonical AAG PAM and 5 to 10 nt matching the seed region of a crRNA from the CRISPR-I array (spacers 1, 3, 4, and 8) (Fig. 2B; see also Fig. S2C and Table S2). The strongest binding events were associated with spacer 8 of CRISPR-I (“sp1.8”) (Fig. 2B; see also Fig. S2C). To confirm that Cascade binding events were due to association with endogenous crRNAs, we repeated the ChIP-seq experiment in cells lacking the CRISPR-I array and in cells lacking the CRISPR-II array. Deletion of CRISPR-II had little effect on the profile of Cascade binding (Fig. 2C; see also Table S1). In contrast, deletion of CRISPR-I resulted in loss of Cascade binding to almost all sites bound in wild-type cells (Fig. 2D; see also Table S1). Instead, low-level binding of Cascade was observed at a small number of sites that were associated with a weakly enriched sequence motif corresponding to a perfect PAM and 8 nt matching the seed region of spacer 2 of CRISPR-II (Fig. S2D; see also Fig. S3 and Table S2).

10.1128/mBio.02100-17.3 FIG S3  Spacer 2 of CRISPR-II directs Cascade binding in cells lacking CRISPR-I. The figure shows an enriched sequence motif associated with Cascade binding sites in cells expressing only endogenous crRNAs, where CRISPR-I is deleted (LC077). The motif is associated with CRISPR-II spacer 2, as indicated. The likely PAM sequence is also indicated. The number of identified motifs and the MEME E value are shown. Download FIG S3, PDF file, 0.1 MB. Copyright © 2018 Cooper et al. 2018 Cooper et al. This content is distributed under the terms of the Creative Commons Attribution 4.0 International license.

### CRISPR-I spacer 8 is the major determinant of off-target Cascade binding in cells expressing endogenous crRNAs.

**FIG 3:**
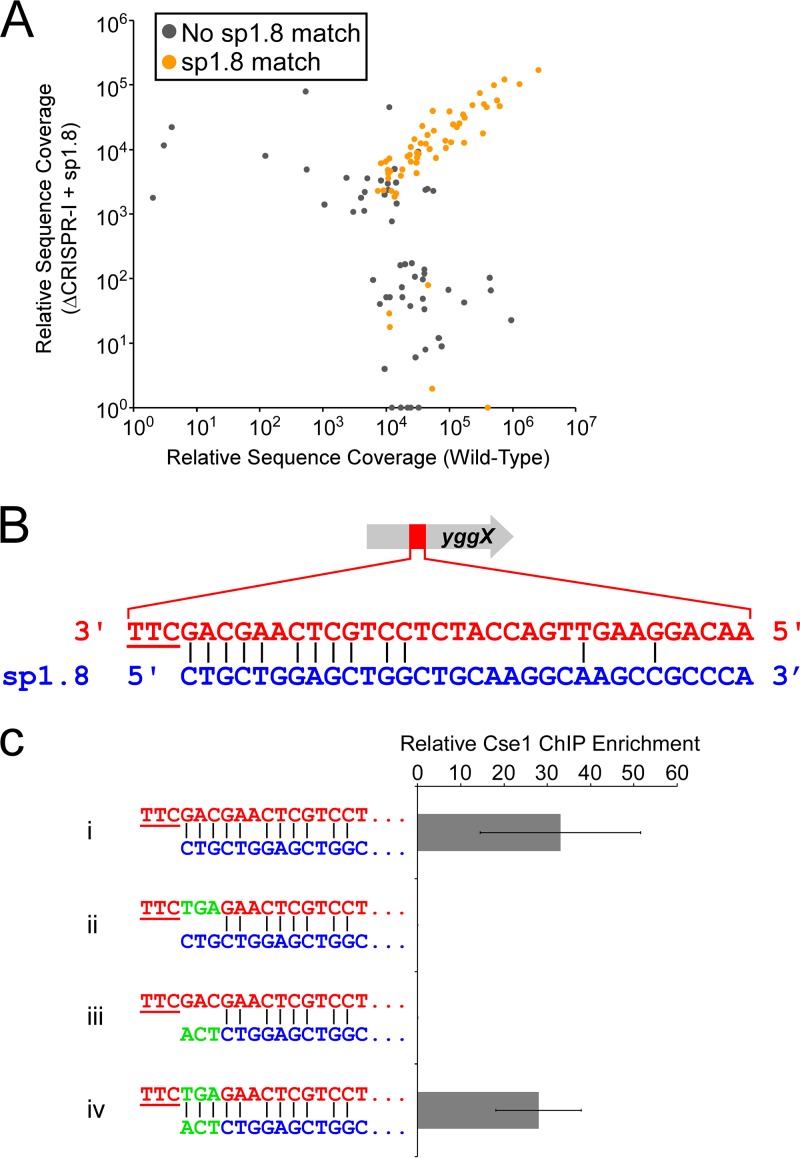
CRISPR-I spacer 8 is responsible for the majority of Cascade binding in cells expressing only endogenous crRNAs. (A) Comparison of Cse1-FLAG_3_ binding events in cells with both CRISPR arrays intact (AMD543) and in cells with CRISPR-I deleted (LC077) that express CRISPR-I spacer 8 from a plasmid (pLC008). Sequence read coverage is shown for all ChIP-seq peaks identified for either strain. ChIP-seq peaks associated with the CRISPR-I spacer 8 motif (first motif listed in Fig. 2B) are shown in orange. (B) Predicted base-pairing interaction between CRISPR-I spacer 8 and a protospacer within *yggX*. The PAM is underlined. (C) ChIP-qPCR measurement of Cse1 binding at wild-type (i and iii; AMD566) and mutant (ii and iv; LC099) protospacers in *yggX* for cells expressing wild-type (i and ii; pLC008) or mutant (iii and iv; pLC010) CRISPR-I spacer 8 from a plasmid. The mutations in spacer 8 restored base-pairing potential with the mutant protospacer, as indicated. Values represent averages of results from three independent replicate experiments. Error bars show 1 standard deviation from the mean.

Our data suggested that the majority of Cascade binding associated with endogenous crRNAs is due to CRISPR-I and that the dominant spacer from CRISPR-I is sp1.8. To confirm this, we measured the levels of Cascade binding by ChIP-seq in cells lacking CRISPR-I but carrying a plasmid expressing sp1.8 crRNA. Note that the plasmid-expressed sp1.8 crRNA differs from sp1.8 at the last two nucleotides of the spacer. However, these mismatches are not expected to affect Cascade binding (23, 38). Most of the Cascade binding sites that we observed were identical to those seen in cells expressing both CRISPR arrays or in cells expressing only CRISPR-I (Fig. 3A; see also Table S1) and corresponded to regions containing strong matches to sp1.8 (the orange dots in Fig. 3A correspond to regions containing a match to the sp1.8 motif shown in Fig. 2B). As expected, and unlike the results determined with cells expressing CRISPR-I, we detected only a single strongly enriched sequence motif (Fig. S4A; see also Table S2). This motif, as expected, corresponds to an AAG PAM and to 9 nt matching the seed region of sp1.8 (Fig. S2C). We also detected a weakly enriched sequence motif (Fig. S4B and Table S2) that corresponds to an AAG PAM and the 11 nt immediately downstream of the second repeat on the plasmid expressing the sp1.8 crRNA. This was likely due to formation of a noncanonical crRNA that consisted of the sequence between the second repeat and the transcription terminator (Fig. S2E). A transcription terminator hairpin has previously been shown to function analogously to repeat sequences in the E. coli crRNAs (39).

10.1128/mBio.02100-17.4 FIG S4  Sequence motifs associated with Cascade binding in cells expressing CRISPR-I spacer 8 from a plasmid. (A) Sequence of the most strongly enriched motif, as identified by MEME, in ΔCRISPR-I cells (LC077) expressing CRISPR-I spacer 8 from a plasmid (pLC008). The motif is associated with CRISPR-I spacer 8, as indicated. The likely PAM sequence is also indicated. The number of identified motifs and the MEME E value are shown. (B) The second enriched sequence motif, as identified by MEME, in ΔCRISPR-I cells (LC077) expressing CRISPR-I spacer 8 from a plasmid (pLC008). The motif is associated with the sequence immediately downstream of the second repeat on the crRNA plasmid, as indicated. Download FIG S4, PDF file, 0.1 MB. Copyright © 2018 Cooper et al. 2018 Cooper et al. This content is distributed under the terms of the Creative Commons Attribution 4.0 International license.

The most highly enriched Cascade target region in cells with CRISPR-I and in cells expressing sp1.8 crRNA was inside the *yggX* gene. We identified a sequence in this region with an AAG PAM and with matches to positions 1 to 5 and positions 7 to 10 of sp1.8 (Fig. 3B). We used targeted ChIP-quantitative PCR (ChIP-qPCR) to measure Cascade binding to this site in cells lacking CRISPR-I but carrying a plasmid expressing sp1.8 crRNA (with mismatches to sp1.8 at the last two nucleotide positions, as described above). We compared binding of Cascade to *yggX* in wild-type cells and in cells where the putative protospacer was mutated in the region predicted to bind the sp1.8 crRNA seed. As expected, we observed greatly reduced Cascade binding at the mutated site relative to the wild-type site. Similarly, we observed greatly reduced Cascade binding at the wild-type site when we expressed a mutant sp1.8 with changes in the seed region (Fig. 3C). However, when we combined the mutant spacer with the mutant protospacer, base-pairing potential was restored and we observed wild-type levels of Cascade binding (Fig. 3C). We conclude that sp1.8 is the major determinant for off-target Cascade binding in cells expressing endogenous crRNAs.

### Off-target Cascade binding events do not affect local gene expression.

Cascade binding events can lead to transcription repression by preventing the initiation of RNA polymerase binding to a promoter or by acting as a roadblock to elongating RNA polymerase within a transcription unit (38, 40). To determine if off-target events driven by endogenous spacers affect local gene expression, we measured global RNA levels using transcriptome sequencing (RNA-seq) in Δ*cas3* cells with other *cas* genes constitutively expressed, and with either intact CRISPR arrays or a ΔCRISPR-I deletion. We detected few differences in RNA levels between the two strains (Table S3), and none of the differences corresponded to genes within 1 kb of a Cascade binding site identified by ChIP-seq. We conclude that off-target binding by a Cas3-deficient complex does not impact local gene expression.

10.1128/mBio.02100-17.7 TABLE S3  Analysis of RNA-seq data. Download TABLE S3, XLSX file, 0.5 MB. Copyright © 2018 Cooper et al. 2018 Cooper et al. This content is distributed under the terms of the Creative Commons Attribution 4.0 International license.

### No evidence for RNA targeting by E. coli Cascade.

A recent report suggested that Cascade binding to RNAs in Pseudomonas aeruginosa, which has a type I-F system, leads to Cas3-mediated degradation of the target RNA (41). Moreover, that study suggested that only 8 nt of sequence complementarity between the crRNA and target RNA and a flanking 5′-GGA-3′ sequence are required to recruit Cas3. This is similar to the sequence requirement for off-target binding to DNA sites (Fig. 1 and 2), suggesting that Cascade could target many endogenous RNAs (42). To determine whether the E. coli type I-E CRISPR-Cas system targets RNA in a similar way, we measured global RNA levels using RNA-seq in cells expressing *cas3* from a plasmid and all other *cas* genes from their chromosomal loci, with either intact CRISPR arrays or a ΔCRISPR-I deletion. We compared these data to the data described above for Δ*cas3* cells with either intact CRISPR arrays or a ΔCRISPR-I deletion. We reasoned that targeted RNAs would be less abundant in cells expressing both Cas3 and CRISPR-I. However, we detected only two genes, *ykgE* and *glpD*, for which RNA levels were significantly lower in the *cas3*-positive (*cas3*^+^) CRISPR-I^+^ strain than in the strains lacking either or both of *cas3* and CRISPR-I (Table S3). Only one of these genes (*glpD*) contains an 8-nt sequence complementary to the 3′ end of a spacer in CRISPR-I (spacer 4; we included the predicted 5′ untranslated region [UTR] in the search for both RNAs). Given the length of the two genes, finding an 8-nt match by chance is not unlikely. Moreover, three other genes contain the same 8-nt match to spacer 4, with the same 3-nt flanking sequence, but these genes did not have the RNA profile expected for a Cascade target. Thus, our data strongly suggest that the type I-E CRISPR-Cas system in E. coli does not target RNA using a mechanism similar to that described for the type I-F system in P. aeruginosa.

### Off-target Cascade binding is not associated with interference.

**FIG 4:**
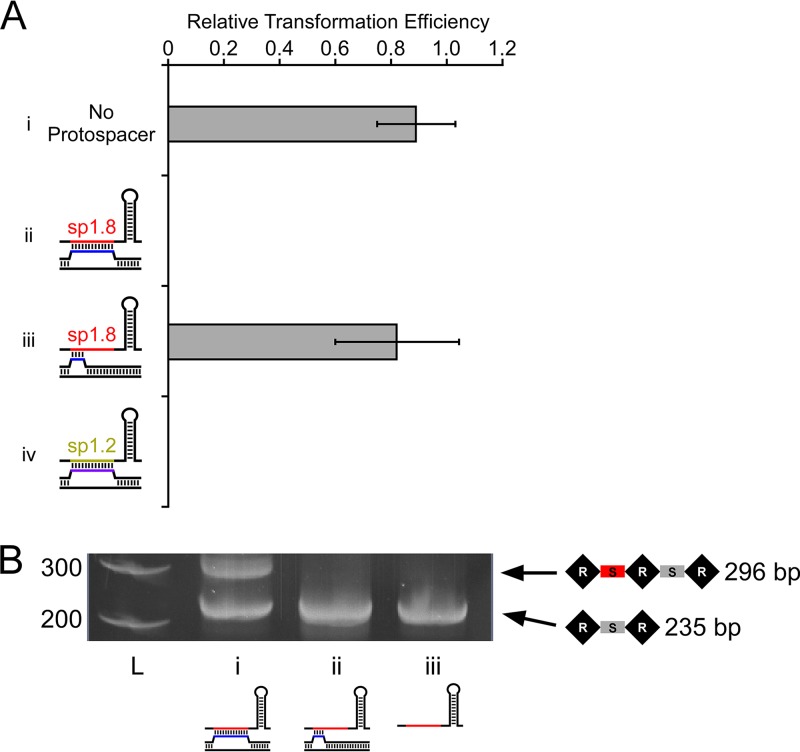
Off-target Cascade binding events are not associated with interference or primed adaptation. (A) Relative levels of efficiency of transformation of a *cas3*-expressing plasmid (pAMD191) into LC103 cells expressing spacer 8 from a native CRISPR-I array and containing (i) empty pBAD24 (“No Protospacer”), (ii) a plasmid with a protospacer that base pairs perfectly with spacer 8 (pLC022), (iii) a plasmid with a protospacer that has only partial base pairing with CRISPR-I spacer 8 (pLC021; the protospacer sequence matches the off-target Cascade binding site in *yggX*), or (iv) a plasmid with a protospacer that base pairs perfectly with CRISPR-I spacer 2 (pLC057). Note that crRNAs were expressed from the chromosome, since both CRISPR arrays are intact in these strains. Transformation efficiency was calculated relative to that of empty pBAD33, as described in Materials and Methods. Values represent averages of results from three independent replicate experiments. Error bars show 1 standard deviations from the means. The calculated transformation efficiency for protospacers ii and iv was 0, but the limit of detection in this assay was 3e^−5^. (B) PCR amplification of the start of the CRISPR-II array to detect primed adaptation in cells expressing CRISPR-I spacer 8 (AMD536) and *cas3* (pAMD191) and with (i) a protospacer that base pairs perfectly with CRISPR-I spacer 8 (pLC022), (ii) the protospacer from *yggX* that has only partial base pairing with CRISPR-I spacer 8 (pLC021), or (iii) empty vector (pBAD24). L, molecular weight ladder, with marker sizes indicated. The expected PCR product sizes are indicated.

Previous studies have suggested that extensive mismatches at the PAM-proximal end of the spacer/protospacer prevent interference (16, 35). To determine whether off-target Cascade binding events lead to interference, we constructed a Δ*yggX* Δ*cas3* strain expressing all other *cas* genes, with both CRISPR arrays intact. We introduced a plasmid expressing *cas3* or an equivalent empty vector. We then transformed these strains with a plasmid containing the off-target protospacer from *yggX* that is an imperfect match to sp1.8, with an equivalent plasmid with a protospacer that is a perfect match to sp1.8, with a plasmid with a protospacer that is a perfect match to CRISPR-I spacer 2 (“sp1.2”), or with empty vector. We reasoned that the number of viable transformants for plasmids with interference-proficient protospacers would be low for cells expressing Cas3, since interference would cause loss of the protospacer-containing plasmid, leading to killing by the antibiotic selection. In contrast, the number of viable transformants for plasmids with interference-deficient protospacers, or cells not expressing Cas3, should be high. We measured the transformation efficiency for plasmids containing each of the protospacers in cells with a Cas3-expressing plasmid or an equivalent empty vector. The efficiency of interference was calculated using the ratio of transformation efficiency for cells with Cas3 to that for cells without Cas3. As expected, the experiment performed with the protospacer that perfectly matched sp1.8 resulted in highly efficient interference. Similarly, the experiment performed with the protospacer that perfectly matched sp1.2 resulted in highly efficient interference. We conclude that sp1.2 is efficiently assembled into Cascade, despite the lack of chromosomal off-target binding events detected by ChIP-seq. In contrast, the protospacer with the native *yggX* sequence (i.e., with an imperfect match to sp1.8) resulted in no detectable interference (Fig. 4A). We conclude that off-target Cascade binding events do not cause interference.

### Off-target Cascade binding is not associated with primed adaptation.

Protospacers with multiple mismatches to a crRNA can still cause primed adaptation (23), and a recent study concluded that Cascade can bind to a protospacer with extensive mismatches, including in the seed region or at the PAM-distal end, and that these binding events cause primed adaptation (35). To test whether off-target Cascade binding is sufficient for primed adaptation, we used the strains described above that contained a plasmid with a protospacer that is either an imperfect or a perfect match to sp1.8. We then introduced a plasmid with an inducible copy of *cas3*, under noninducing conditions, to avoid interference. Following induction of *cas3* expression, we harvested cells and used PCR amplification of the 5′ end of the CRISPR-II array to determine whether new spacers had been acquired because of primed adaptation. We observed robust primed adaptation for the protospacer with a perfect match to sp1.8 but no detectable adaptation for the off-target protospacer with an imperfect match to sp1.8 (Fig. 4B). We conclude that off-target Cascade binding events do not lead to primed adaptation.

### Strong Cascade binding to protospacers with extensive mismatches at the crRNA PAM-distal end.

**FIG 5:**
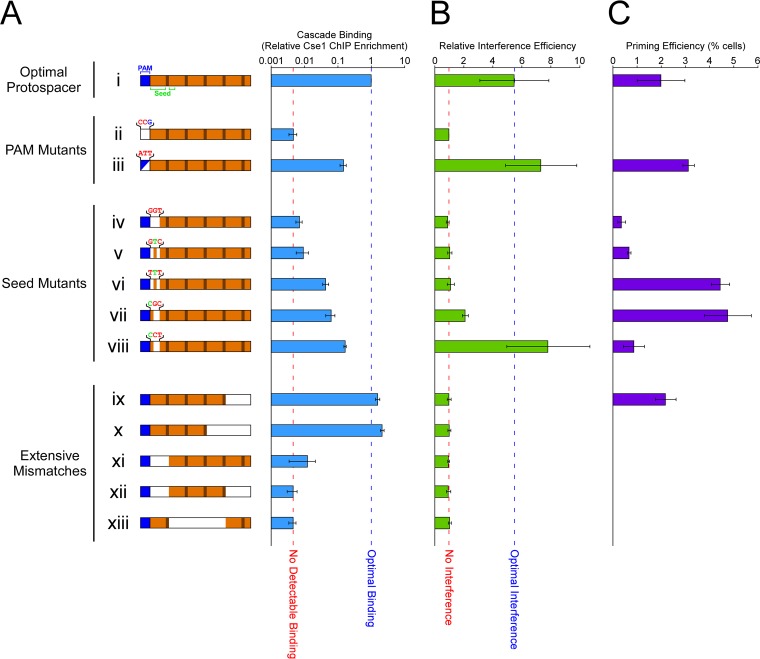
Assessment of Cascade binding, interference, and primed adaptation for a panel of protospacer variants. (A) Relative Cse1 association (in strain LC099, which expresses CRISPR-I spacer 8 crRNA from the native CRISPR-I array) for each of 13 protospacer variants. The protospacer variants are (i) an optimal protospacer that has a perfect match to CRISPR-I spacer 8 and an AAG PAM (pLC023); (ii) CCG PAM (pLC027); (iii) ATT PAM (pLC029); (iv) mismatches at positions 1 to 3 (pLC031; the wild type is CTG); (v) mismatches at positions 1 and 3 (pLC033); (vi) mismatches at positions 1 and 3 (pLC035); (vii) mismatches at positions 2 and 3 (pLC032); (viii) mismatches at positions 2 and 3 (pLC034); (ix) mismatches across positions 25 to 32 (pLC024); (x) mismatches across positions 19 to 32 (pLC025); (xi) mismatches across positions 1 to 6 (pLC026); (xii) mismatches across positions 1 to 6 and positions 25 to 32 (pLC028); and (xiii) mismatches across positions 7 to 24 (pLC030). Values represent averages of results from five independent replicate experiments. Error bars show 1 standard deviation from the mean. (B) Relative efficiency of interference (see Materials and Methods for details) for each of the indicated protospacer variants (pLC023 to pLC035). Values represent averages of results from three independent replicate experiments. Error bars show 1 standard deviation from the mean. (C) Levels of primed adaptation in AMD688 cells expressing CRISPR-I spacer 8 from a native CRISPR-I array and *cas3* (pAMD191) and with each of the 13 indicated protospacer variants (pLC023 to pLC035). Adaptation was measured by monitoring the conversion of a *yfp* reporter construct into the actively expressed state (see Materials and Methods for details). Values shown represent the percentages of the population that converted to YFP^+^. Background levels of adaptation were detected for variants ii, x, xi, xii, and xiii.

To further delineate the protospacer sequence requirements for Cascade binding, interference, and primed adaptation, we constructed 13 variants of a protospacer that matches sp1.8. We selected sp1.8 because it elicits robust Cascade binding, interference, and primed adaptation (Fig. 3 and 4). The protospacer variants (Fig. 5A) included the following: variant I, the “optimal” protospacer, with full sequence complementarity and an optimal, AAG PAM; variants ii and iii, with nonoptimal PAMs, including CCG, which is expected to completely abolish Cascade binding (34), and ATT, a suboptimal sequence previously shown to cause primed adaptation but not detectable interference (31); variants iv to viii, with two or three mismatches in the first three positions of the seed; and variants ix to xiii, with stretches of ≥6-nt mismatches at various positions within the protospacer.

We pooled cells containing each of the protospacer variants. We used ChIP of Cse1-FLAG_3_ in Δ*cas3* cells to measure association of Cascade with all protospacers within the pool (see Materials and Methods). As expected, the protospacer with a CCG PAM (variant ii) had far less Cascade association than did the optimal protospacer (variant i) (Fig. 5A). We presume that the level of ChIP signal for the protospacer with the CCG PAM (variant ii) represents the background of this experiment. The protospacer with a suboptimal, ATT PAM (iii) showed reduced Cascade binding relative to the optimal protospacer (variant i), but the level was well above the experimental background (Fig. 5A). Similarly, mismatches in the seed region (variants iv to viii) resulted in a reduction in Cascade association (Fig. 5A). Our data for PAM and seed mutants are consistent with earlier studies showing that these sequences are important for Cascade binding (17, 29, 30, 34).

Mismatches in the protospacer at positions 1 to 6 (variants xi and xii) or positions 7 to 20 (variant xiii) abolished Cascade binding (Fig. 5A). This is consistent with the observation from our ChIP-seq data that sequence matches in positions 1 to 8 appear to be required for Cascade binding to off-target sites using sp1.8 (Fig. 2B; see also Fig. S4A). Strikingly, mismatches across positions 25 to 32 (variant ix) or positions 19 to 32 (variant x) did not reduce the Cascade association relative to that seen with the optimal protospacer (variant i) (Fig. 5A). Thus, our data confirm that PAM-proximal sequence is necessary for Cascade binding, while PAM-distal sequence is insufficient for Cascade binding.

### Extensive crRNA-protospacer base pairing is required for interference and primed adaptation.

We next determined which of the protospacer variants lead to interference. Using a modification of a previously described assay (see Materials and Methods) (23, 31), we measured the level of interference with a plasmid target for each of the 13 protospacers, using Δ*cas1* cells that cannot acquire new spacers; primed adaptation cannot contribute to the level of interference in these cells. As expected, the optimal protospacer (variant i) was associated with robust levels of interference, whereas protospacer variants that do not bind Cascade (variants ii, xi, xii, and xiii; Fig. 5A) were not associated with detectable interference (Fig. 5B). Protospacers with PAM and seed variants that showed reduced but not abolished Cascade binding (variants iii, vi, vii, and viii; Fig. 5A) were associated with a range of interference levels that correlate well with the level of Cascade binding. Seed mutants with stronger defects in binding exhibited no detectable interference. However, the ability of protospacers to cause interference did not always correlate with the level of Cascade association. Specifically, we detected no interference for either of the protospacer variants with mismatches only at the PAM distal end (variants ix and x; Fig. 5B), even though those protospacers bound Cascade at least as well as the optimal protospacer (Fig. 5A).

Previous studies have proposed that some protospacers with suboptimal PAMs or mismatches in the seed region are not subject to detectable interference but do cause primed adaptation (21, 23, 28, 31, 35). We determined whether the 13 protospacer variants caused primed adaptation in a plasmid context. We used a highly sensitive assay for adaptation that relies on expression of a *yfp* reporter gene that is encoded immediately upstream of a CRISPR array. Translation is terminated upstream of *yfp* in cells without newly acquired spacers, whereas acquisition of one spacer/repeat puts *yfp* back in frame (43), causing cells to fluoresce. We introduced an inducible copy of *cas3* into cells with an intact CRISPR-I array, and containing each of the protospacers on a high-copy-number plasmid and the *yfp* reporter construct. We then induced expression of *cas3* and measured the level of primed adaptation using flow cytometry. In this experiment, we expected the sp1.8 crRNA from the native CRISPR-I array to cause primed adaptation. We detected primed adaptation for all protospacers associated with detectable interference (variants i, iii, vii, and viii; Fig. 5C), although the level of adaptation was lower for two of the constructs with the highest levels of interference (variants I and viii). This was likely due to high levels of interference reducing the amount of substrate available for adaptation (33). In contrast, we observed no adaptation for protospacers that do not bind Cascade (variants ii, xi, xii, and xiii; Fig. 5C). Strikingly, we observed primed adaptation for four protospacers that were not associated with detectable interference (Fig. 5C). Three of these protospacers have seed mismatches and exhibited the lowest levels of Cascade binding (Fig. 5A; variants iv, v, and vi). The other protospacer has mismatches across positions 25 to 32 (variant ix). Thus, for these protospacers, we detected Cascade binding and primed adaptation but were unable to detect interference. For the protospacer with mismatches across positions 19 to 32 (variant x), we detected no primed adaptation. Thus, for this protospacer, we detected robust Cascade binding but no primed adaptation or interference. Overall, our data suggest that extensive crRNA-protospacer base pairing from the PAM-proximal end is required for both interference and primed adaptation and that primed adaptation is a more sensitive assay of CRISPR-Cas function than interference.

## DISCUSSION

### Base pairing in the seed region together with an AAG PAM is sufficient for Cascade to bind DNA.

No previous studies have measured Cascade binding to protospacer DNA *in vivo*. Our ChIP data indicate that an AAG PAM and as little as 5 nucleotides of base pairing at the start of the seed region are sufficient for E. coli Cascade to bind DNA targets. The sequence requirements for protospacer binding in type II systems are similarly relaxed (44–46). The affinity of Cascade for a protospacer increases as the extent of base pairing increases, but maximal affinity occurs with no more than an 18-bp match at the PAM-proximal end (Fig. 5A). Analysis of Cascade interactions with DNA *in vitro* suggests that Cascade associates for brief periods with PAM-containing sequences and does so for longer periods if there is partial base pairing in the seed region (28). Our data support these observations, although we did not detect ChIP signal at PAM sequences that lack seed matches, suggesting that seed base pairing contributes more to Cascade association *in vivo*. Consistent with this suggestion, the difference between the ChIP signals for off-target sites and on-target sites is considerably less than the difference between dwell times *in vitro* (28), although the use of cross-linking in ChIP may also contribute to this difference, since cross-linking “locks” Cascade on the DNA.

### AAG is the optimal PAM in E. coli*.*

Three previous studies proposed that AAG, GAG, TAG, AGG, and ATG are optimal PAMs in E. coli (23, 31, 47), while another study suggested that AAG, ATG, and GAG PAMs were associated with moderately higher-affinity Cascade binding than an AGG PAM (34). Our data clearly indicate that AAG is the optimal PAM for off-target sites, with most off-target Cascade binding events being associated with an AAG PAM. Specifically, 65% of the Cascade binding sites associated with a detectable motif have an AAG PAM for the crRNAs targeting *lacZ* and *araB* and the plasmid-expressed sp1.8 crRNA. Moreover, off-target Cascade binding events with higher enrichment scores, suggestive of higher Cascade affinity, were more likely to be associated with an AAG PAM than Cascade binding events with lower enrichment scores (76% versus 61% for the top 20% and bottom 80% of bound regions, respectively, after sorting by Cse1 enrichment level). We hypothesize that the dependence on the PAM for Cascade binding is increased in situations where base pairing occurs only in the seed region. According to this model, complete or nearly complete base pairing between the crRNA and protospacer would reduce the requirement for an optimal PAM, obscuring the differences in PAM affinity. This would explain why previous studies suggested that there are at least three optimal PAMs (23, 31, 34, 47).

### Defining the crRNA seed region.

The seed region of a crRNA has been previously defined as consisting of positions 1 to 5 and positions 7 to 8, with position 1 being immediately adjacent to the PAM (29). However, our data suggest that the lengths of the seed region differ between crRNAs, since we observed off-target binding with some crRNAs that required base pairing in positions 1 to 5, whereas off-target binding for other crRNAs required base pairing up to position 9 (Fig. 1 and 2; see also Fig. S3 and S4 in the supplemental material). We propose that the crRNA sequence determines the length of the seed region and that this reflects the initial binding mode, prior to extended base pair formation. Every sixth position of the crRNA is flipped out in the Cascade-protospacer complex and hence does not contribute to base pairing (15, 48, 49). Consistent with this, position 6 is substantially less important for off-target binding than positions 1 to 5 (Fig. 1 and 2; see also Fig. S3 and S4). Nonetheless, off-target protospacers had a sequence match to the crRNA at position 6 far more frequently than would be expected by chance (45% for the crRNAs targeting *lacZ* and *araB* and for the plasmid-expressed sp1.8 crRNA; binomial test *P* value = 2.4e^−10^). We hypothesize that the initial binding of Cascade to a protospacer includes base-pairing interactions at position 6 but that the complex rapidly transitions to a conformation in which the sixth position is flipped out of the helix. Our data are consistent with an *in vitro* study of another type I-E system, where position 6 was also shown to contribute to off-target Cascade binding (50). The apparent requirement for a sequence match at position 6 was not consistent across all of the crRNAs that we tested, suggesting that the pathways toward stable seed base pairing differ in a sequence-dependent manner.

### Interference and primed adaptation require extended R-loop formation.

Although binding of Cascade to a DNA target requires relatively little sequence identity, our data indicate that robust interference and primed adaptation require at least 18 to 25 bp, beginning in the seed region. This is consistent with *in vitro* data showing that near-complete R-loop formation is required to license Cas3 activity (16). Thus, although Cascade binds DNA promiscuously, functional binding occurs with high specificity. Our data support a previously proposed model in which extended R-loop formation triggers a conformational change in Cascade at the PAM-distal end of the spacer, which is then transmitted, presumably through Cse2, to PAM-associated Cse1 (16, 51). This change in Cse1 conformation then recruits Cas3 or activates the nuclease activity of Cas3 or both, as suggested by a recent structural study (51).

### Evidence that interference and primed adaptation are obligately coupled processes.

Primed adaptation was initially proposed to be an alternative pathway to interference, with optimal PAM/seed sequences leading to interference and suboptimal sequences leading to primed adaptation (21, 23, 28, 31, 35, 52). However, primed adaptation has been observed in situations where interference occurs (Fig. 5, variants i, iii, vii, and viii) (22, 24, 32, 33), suggesting that primed adaptation and interference can be coupled processes and supporting the idea that primed adaptation represents a positive-feedback loop (22). While these data show that primed adaptation and interference can occur at the same time at a population level, they do not necessarily indicate that individual primed adaptation and interference events are coupled. Moreover, while it has been proposed that interference and primed adaptation are obligately coupled (53), this has not been tested. There are many examples where primed adaptation has been observed in the absence of detectable interference (21, 23, 31, 32, 35, 52). However, this can be explained by the fact that primed adaptation is likely to be a more sensitive assay of CRISPR-Cas function than interference, as there would be detectable primed adaptation but not detectable interference in cells where target DNA replication outpaces interference (53). Our data are consistent with a model in which primed adaptation and interference are coupled processes: seed mismatches reduce Cascade binding, and we observed a corresponding effect on interference and primed adaptation, with primed adaptation being a more sensitive assay for CRISPR-Cas function (Fig. 5). The only exception to this trend is the seed mismatch with the highest level of binding (Fig. 5, variant viii), which has relatively low levels of primed adaptation. However, very efficient interference with this variant likely depletes the substrate for primed adaptation (33). Unexpectedly, we observed primed adaptation in the absence of detectable interference for a protospacer with mismatches across positions 25 to 32 (Fig. 5, variant ix). We propose that this degree of mismatch at the 3′ end of the crRNA greatly reduces, but does not abolish, the isomerization of Cascade into the “active” state that recruits/activates Cas3.

### Extensive, inert, off-target binding of Cascade.

Cascade has many off-target binding sites due to its ability to bind DNA with low sequence specificity. Consequently, the endogenous crRNAs transcribed from the bacterial genome result in extensive off-target binding, even in the absence of an on-target site. Since off-target binding does not involve extended R-loop formation, it has no deleterious effects on genome integrity. We also observed no impact on transcription associated with any of the off-target binding events, despite the fact that targeted Cascade binding is known to repress transcription by occluding promoters or acting as a roadblock for elongating RNA polymerase (38, 40). Transcription repression by Cascade is considerably weaker when targeting within a transcribed region (i.e., acting as a roadblock) (38). Given that the location of off-target Cascade binding sites is essentially random with respect to genome organization, and that genes make up ~90% of the E. coli genome, off-target Cascade binding is expected to be primarily intragenic. This may partly explain the lack of impact on transcription. Moreover, a recent study showed that the level of repression by Cascade occlusion of a promoter is greatly reduced with as few as 6 bases mismatched at the PAM-distal end of the spacer/protospacer (54), suggesting that even intergenic off-target Cascade binding sites would be transcriptionally inert. We propose that incomplete R-loop formation results in an unstable Cascade-DNA complex with a relatively high rate of dissociation, such that it cannot compete effectively with initiating or elongating RNA polymerase. Consistent with this model, stable association of Cascade with DNA *in vitro* has been shown to require near-complete R-loop formation (18). We conclude that type I CRISPR-Cas systems have evolved to tolerate off-target binding driven by the endogenous crRNAs and that they are functional only at on-target sites. Given the length of crRNA spacers in type I systems, there is no expectation of complete or near-complete spacer-protospacer base pairing by chance. Note that self-targeting by type I CRISPR-Cas systems has been described previously, but these would be considered “on-target” events, likely caused by acquisition of spacers from the chromosome. As expected for spacers with perfect sequence complementarity, these self-targeting crRNAs are typically functional in gene regulation and interference (36, 37, 55).

### Not all crRNAs are created equal.

The E. coli genome encodes at least 19 crRNAs, and yet our data suggest that only four crRNAs contribute to off-target binding of Cascade. All four of these crRNAs are encoded in the CRISPR-I array, and the majority of off-target binding is driven by just one, sp1.8. The lack of off-target binding driven by CRISPR-II crRNAs is likely due to weak transcription of this array, which is repressed by H-NS (56). In contrast, the CRISPR-I array is likely cotranscribed with the upstream *cas* genes, which are strongly transcribed in the strain used in this study. The preference for specific spacers within CRISPR-I cannot be explained by differences in expression levels, since the crRNAs are transcribed as a single RNA. Rather, biases in spacer usage are more likely due to differential assembly of specific crRNAs into Cascade. Consistent with this, a previous study surveyed crRNAs associated with Cascade. Spacers 2, 4, and 8 from CRISPR-I represented 68% of the Cascade-associated crRNAs (7). The cause of this bias is unclear but may be due in part to differences in the RNA secondary structure between spacers, which could impact the efficiency of RNA processing by Cas6e. Consistent with this, the RNA secondary structure of repeat sequences and associated processing by Cas6 have been shown to be impacted by spacer sequences in the type I-D system of *Synechocystis* sp. strain PCC 6803 (57). Nonetheless, it is likely that other factors influence the level of off-target binding, since the relative levels of association of crRNAs for spacers 2, 4, and 8 with Cascade are likely to be similar (7) and since sp1.2 causes efficient interference (Fig. 4A), but sp1.8 drives a disproportionately high level of off-target binding relative to sp1.2. Strikingly, there are many more chromosomal sequence matches to the seed sequence of sp1.8 coupled with an AAG PAM than for any other spacer (see Table S4 in the supplemental material). This is likely due to the fact that the sequence from position −1 (i.e., the last base of the PAM) to +8 of sp1.8 differs from the canonical Chi site sequence (5′-GCTGGTGG-3′) (58) by a single nucleotide; Chi sites are strongly enriched in the E. coli K-12 genome (59). Moreover, positions 3 to 7 of sp1.8 (5′-GCTGG-3′) are a perfect match to a sequence that is strongly enriched in the E. coli K-12 genome (59). We conclude that extensive off-target binding driven by sp1.8 is likely due to a combination of a high level of association with Cascade and a relatively high level of abundance of potential binding sites in the genome.

10.1128/mBio.02100-17.8 TABLE S4  Numbers of potential off-target chromosomal binding sites for spacers in the CRISPR-I array. Download TABLE S4, PDF file, 0.03 MB. Copyright © 2018 Cooper et al. 2018 Cooper et al. This content is distributed under the terms of the Creative Commons Attribution 4.0 International license.

## MATERIALS AND METHODS

### Strains and plasmids.

All strains, plasmids, oligonucleotides, and purchased, chemically synthesized double-stranded DNA (dsDNA) fragments are listed in Table S5 in the supplemental material. All strains used were derivatives of MG1655 (59). CB386 has been previously described (38). CB386 contains a chloramphenicol resistance cassette in place of *cas3*. We removed this cassette using Flp recombinase, expressed from plasmid pCP20 (60), to generate strain AMD536. Epitope-tagged strains AMD543 and AMD554 (Cse1-FLAG_3_ and FLAG_3_-Cas5, respectively) are derivatives of CB386 and were generated using the previously described FRUIT method of recombineering (61). Cse1 was C-terminally tagged in AMD543 by inserting a FLAG_3_ tag immediately upstream of codon 495 using oligonucleotides JW6364 and JW6365. Tagging of Cse1 resulted in an 8-amino-acid C-terminal truncation. We predicted on the basis of phylogenetic comparisons and of structural data (49) that this truncation would not impact the function of Cse1. Cas5 was N-terminally tagged in AMD554 by inserting FLAG_3_ using oligonucleotides JW6272 and JW6273. LC060 is a derivative of AMD536 and was generated using (i) FRUIT (61) with oligonucleotides JW7537-JW7540 to delete the CRISPR-II locus, (ii) P1 transduction of the CB386 (Δ*cas3* P*cse1*)::(Cat::P_J23199_) region, (iii) FRUIT (61) to C-terminally tag Cse1 with FLAG_3_ (as described above for AMD543), and (iv) pCP20-expressed Flp recombinase (60) to remove the *cat* cassette. LC074 is a derivative of AMD536 in which the CRISPR-I array was deleted using FRUIT (61) with oligonucleotides JW7529 and JW7530 and a synthesized dsDNA fragment (gBlock 14148263; Integrated DNA Technologies, Inc.). LC077 is a derivative of LC074 in which Cse1 was C-terminally tagged with FLAG_3_ (as described above for AMD543). AMD566 is a derivative of AMD536 in which Cse1 was C-terminally tagged with FLAG_3_ (as described above for AMD543). LC099 is a derivative of AMD566 in which the off-target binding site for Cascade in *yggX* was mutated using FRUIT (61) with oligonucleotides JW7635 to JW7638. LC103 is a derivative of AMD536 in which the *yggX* gene was replaced with a kanamycin resistance cassette using P1 transduction from the Keio Collection Δ*yggX*::Kan^r^ strain (62). LC106 is a derivative of LC103 with an unmarked, scar-free deletion of *cas1* made using FRUIT with oligonucleotides JW7898 to JW7901. AMD688 is a strain that contains a previously reported *yfp* reporter construct that can be used to measure adaptation levels (43). AMD688 was constructed by P1 transduction of the Δ*cas3*::*cat* cassette from CB386 into MLS1003 (provided by the Lundgren laboratory). The *cat* gene was removed using Flp recombinase, expressed from plasmid pCP20 (60). AMD688 has an intact copy of the CRISPR-I array (cotransduced with the Δ*cas3*::*cat* cassette from CB386) but lacks the CRISPR-II array.

10.1128/mBio.02100-17.9 TABLE S5  Strains, plasmids, oligonucleotides, and chemically synthesized dsDNA fragments used in this study. Download TABLE S5, PDF file, 0.2 MB. Copyright © 2018 Cooper et al. 2018 Cooper et al. This content is distributed under the terms of the Creative Commons Attribution 4.0 International license.

Plasmids that express crRNAs targeting the *lacZ* promoter (pCB380) and *araB* promoters (pCB381) have been described previously (38). All other crRNA-expressing plasmids used were derivatives of pAMD179. pAMD179 was constructed by amplifying a DNA fragment from plasmid pAMD172 (Integrated DNA Technologies, Inc.) using oligonucleotides JW6421 and JW6513. This DNA fragment was cloned into pBAD24 (63) cut with NheI and HindIII (NEB) using the In-Fusion method (Clontech). The inserted fragment contains two repeats from the CRISPR-I array, separated by a stuffer fragment containing XhoI and SacII restriction sites, and an intrinsic transcription terminator downstream of the second repeat. To clone individual spacers, pairs of oligonucleotides were annealed, extended, and inserted using In-Fusion (Clontech) into the XhoI and SacII sites of pAMD179 to generate pLC008 (with oligonucleotides JW6518 and JW7911), pLC010 (with oligonucleotides JW6518 and JW7912), and pAMD189 (with oligonucleotides JW7598 and JW7693). Note that the derivatives of sp1.8 expressed from pLC008 and pLC010 differ from sp1.8 at the last two nucleotide positions to facilitate cloning. These mismatches are not expected to affect crRNA function (23, 38).

pLC021, pLC022, and pLC057 are derivatives of pBAD24 (63) that contain a protospacer matching the off-target Cascade binding site in *yggX* (pLC021), a protospacer with a perfect match to sp1.8 (pLC022), or a protospacer with a perfect match to sp1.2 (pLC057). These plasmids were constructed by annealing and extending pairs of oligonucleotides (JW7913 and JW7914 for pLC021, JW7924 and JW7925 for pLC022, and JW9131 and JW9132 for pLC057) and cloning the resultant DNA fragments into the EcoRV and SphI sites of pBAD24. pAMD191 is a derivative of pBAD33 (63) that expresses *cas3* under arabinose control. To construct pAMD191, *cas3* was amplified by colony PCR using oligonucleotides JW7736 and JW7738. The PCR product was cloned into the SacI and HindIII sites of pBAD33 using In-Fusion (Clontech). All protospacers described in the Fig. 5 legend were cloned into plasmid pLC020, the “preprotospacer plasmid,” which is a derivative of pBAD24 (63). pLC020 was generated by cloning the ~500-bp region upstream of E. coli *thyA* (amplified by colony PCR using oligonucleotides JW8040 and JW8128) and the ~500-bp region downstream of E. coli *thyA* (amplified by colony PCR using oligonucleotides JW8042 and JW8043) into the EcoRI site of pBAD24 using In-Fusion (Clontech), simultaneously generating a new EcoRI site between the upstream and downstream regions of *thyA*. The *thyA* gene was then amplified by colony PCR using a universal forward primer (oligonucleotide JW8129) and each of 13 reverse primers (oligonucleotides JW8130, JW8139, JW8145, JW8169, JW8499 to JW8502, and JW8675 to JW8679) containing the 13 protospacer variants described in the Fig. 5 legend The resulting PCR products were cloned into the EcoRI site of the pBAD24 derivative using In-Fusion (Clontech) to generate plasmids pLC023 to pLC035 (see Table S5 for details). Note that plasmids pLC024 and pLC025 differ from pLC023 and from pLC026 to pLC035 at the nucleotide position immediately adjacent to the protospacer, on the PAM-distal side. Differences at this nucleotide position are not expected to affect Cascade binding, interference, or primed adaptation.

### ChIP-qPCR.

Cells were grown overnight in LB and subcultured in LB supplemented with 0.2% arabinose and 100 µg/ml ampicillin at 37°C with aeration to an optical density at 600 nm (OD_600_) of ~0.6. AMD566 and LC099 were used with either pLC008 or pLC010 for ChIP-qPCR. ChIP-qPCR was performed as described previously (64), except that 2 µl anti-FLAG M2 monoclonal antibody (Sigma) and 1 µl anti-σ^54^ monoclonal antibody (NeoClone) were included and processed simultaneously in the immunoprecipitation step. qPCR was performed using oligonucleotides JW7490 to JW7491 (amplifying the off-target site in *yggX*) and JW7922 to JW7923 (amplifying the region upstream of *hypA*). Since σ^54^ is known not to bind within *yggX* (65), we were able to normalize binding of Cse1 within *yggX* to the binding of σ^54^ upstream of *hypA*.

### ChIP-seq.

Strains AMD543, LC060, LC077, AMD543 and AMD554 with pCB380 and pCB381, and LC077 were used for ChIP-seq analysis of Cse1-FLAG_3_ and FLAG_3_-Cas5, except that ampicillin was included only for the experiments involving a crRNA-expressing plasmid and arabinose was included only for the experiments using pLC008. Cells were grown and processed as described for ChIP-qPCR. ChIP-seq was performed in duplicate, following a previously described protocol (66) using 2 µl anti-FLAG M2 monoclonal antibody (Sigma). Sequencing was performed on an Illumina High-Seq 2000 instrument (Next-Generation Sequencing and Expression Analysis Core, State University of New York at Buffalo) or an Illumina Next-Seq instrument (Wadsworth Center Applied Genomic Technologies Core). ChIP-seq data analysis was performed as previously described (67), with reads mapped to the updated MG1655 E. coli genome (GenBank accession number U00096.3). Relative sequence coverage values were calculated by calculating the sequence read coverage at a given genomic location as follows: total number of sequence reads in the run/100,000. Values plotted in Fig. 1A and B and 2A and D represent the maximum values in 1-kbp regions across the genome. *R*^2^ values comparing ChIP-seq data sets were calculated by comparing levels of read coverage at peak centers for all peaks identified for the analyzed data sets. Read coverage at peak centers was determined using a custom Python script. Sequence motifs were identified using MEME (version 4.12.0) (68) with default parameters.

### RNA-seq.

RNA-seq was performed in duplicate with strains AMD536 and LC074, with and without pAMD191. Cells were grown overnight in LB and subcultured in LB (supplemented with 0.2% arabinose and 100 µg/ml ampicillin for experiments involving pAMD191) at 37°C with aeration to an OD_600_ of ~0.6. RNA was purified using a modified hot-phenol method, as previously described (69). Purified RNA was treated with 2 µl DNase (Turbo DNA-free kit; Life Technologies, Inc.) for 45 min at 37°C, followed by phenol extraction and ethanol precipitation. A Ribo-Zero kit (Epicentre) was used to remove rRNA, and strand-specific cDNA libraries were created using a ScriptSeq 2.0 kit (Epicentre). Sequencing was performed using an Illumina Next-Seq instrument (Wadsworth Center Applied Genomic Technologies Core). Differential RNA expression analysis was performed using Rockhopper (version 2.03) with default parameters (70). Differences in RNA levels were considered statistically significant for genes with false-discovery-rate (*q*) values of ≤0.01.

### Plasmid transformation efficiency assay.

LC103 was transformed with either empty pBAD33 or pAMD191 (expresses *cas3*), and these strains were then transformed with pBAD24 (no protospacer) or pLC021 (protospacer with a perfect match to sp1.8) or pLC022 (protospacer with an imperfect match to sp1.8, corresponding to the off-target site in *yggX*) or pLC057 (protospacer with a perfect match to sp1.2). Cells were plated on M9 medium supplemented with 0.2% glycerol, 0.2% arabinose, 100 µg/ml ampicillin, and 30 µg/ml chloramphenicol at 37°C. After overnight growth, colonies were counted, and the relative levels of transformation efficiency were calculated as ratios of transformants for pAMD191-containing cells to transformants for pBAD33-containing cells for each transformed protospacer-containing plasmid.

### PCR to assess primed adaptation.

Primed adaptation was assessed for AMD536 with pAMD191 and either pLC021 or pLC022 (Fig. 4B) and for MG1655, AMD536, AMD543, and AMD544 with pAMD191 and pAMD189 (expresses a self-targeting crRNA; see Fig. S1 in the supplemental material). Cells were grown overnight in LB supplemented with 100 µg/ml ampicillin and 30 µg/ml chloramphenicol at 37°C with aeration and were subcultured the next day in LB supplemented with chloramphenicol and 0.2% arabinose at 37°C with aeration for 6 h. Cells were pelleted from 1 ml of culture by centrifugation, and cell pellets were frozen at −20°C. PCRs were then performed on the cell pellets, amplifying the CRISPR-II array using oligonucleotides JW7818 and JW7819. PCR products were visualized on acrylamide gels.

### Sequence analysis of protospacers from a pooled ChIP library.

LC099 was grown with each of the 13 protospacer variant plasmids (pLC23 to pLC035) overnight in LB supplemented with 100 µg/ml ampicillin. Ten-milliliter subcultures were grown in LB supplemented with 100 µg/ml ampicillin and 0.2% arabinose at 37°C with aeration to an OD_600_ of ~0.6. Three-milliliter volumes from all cultures were combined. ChIP was performed on mixed cultures using 2 µl M2 anti-FLAG monoclonal antibody (Sigma), as previously described (64). A Zymo PCR Clean and Concentrate kit was used to purify ChIP and input DNA. A 50-µl FailSafe (Epicentre) PCR using FailSafe PCR 2× PreMix C and 5.48 ng of ChIP DNA was performed following the manufacturer’s instructions, using oligonucleotide JW8567 and each of oligonucleotides JW8537, JW8556, JW8557, JW8558, JW8559, JW8561, JW8562, JW8563, JW8564, and JW8565 (these incorporate different Illumina indexes). PCR products were purified and concentrated using 0.8× AMPure beads (Beckman Coulter, Inc.; Life Sciences) and sequenced on an Illumina Mi-Seq instrument (Wadsworth Center Applied Genomic Technologies Core). Sequence reads were mapped to each of the 13 protospacer variants using a custom Python script. Relative levels of protospacer abundance in input and ChIP samples for each protospacer were normalized to the total sequence reads. Values for normalized protospacer abundance were further normalized to values from the input sample. Protospacer abundance values are reported relative to those for the optimal protospacer (variant I in Fig. 5).

### Measuring interference for a pooled protospacer library.

Overnight cultures of LC106 strains with each of the 13 protospacer plasmids (pLC023 to pLC035) were grown in LB with 100 µg/ml ampicillin and 30 µg/ml kanamycin. All 13 cultures were combined to make a single subculture (7.7 µl of each overnight culture into a single 10-ml culture). Electrocompetent cells were made and transformed with either empty pBAD33 or pAMD191 (pBAD33-*cas3*). Transformants were plated onto M9 agar supplemented with 0.2% glycerol, 0.2% arabinose, and 30 µg/ml chloramphenicol and were grown overnight at 37°C. Cells were scraped off plates and washed in LB, and protospacers were PCR amplified from cell pellets with oligonucleotide JW8567 and each of oligonucleotides JW8537, JW8558, JW8559, JW8562, JW8563, and JW8566 (these incorporate different Illumina indexes). PCR products were purified and concentrated with 0.8× AMPure beads (Beckman Coulter, Inc.; Life Sciences) and sequenced using an Illumina MiSeq instrument (Wadsworth Center Applied Genomic Technologies Core). Sequence reads were mapped to each of the 13 protospacer variants using a custom Python script. Relative interference efficiency levels were calculated for each protospacer variant by dividing the number of sequence reads from cells transformed with empty pBAD33 by the number of sequence reads from cells transformed with pAMD191 (pBAD33-*cas3*) and normalizing to the value for the protospacer with a CCG PAM (variant ii in Fig. 5).

### Measuring primed adaptation using a yellow fluorescent protein (YFP) fluorescent reporter.

MLS1003 was transformed with each of plasmids LC023 to LC035, and each of the resulting strains was transformed with *cas3*-expressing plasmid pAMD191. Cells were grown overnight at 37°C with shaking in LB supplemented with 100 µg/ml ampicillin and 30 µg/ml chloramphenicol. Cells were subcultured 1:100 for 6 h in LB supplemented with 0.2% l-arabinose and 20 µg/ml chloramphenicol at 37°C with shaking. Cells were pelleted by centrifugation and resuspended in M9 minimal medium in twice the original volume (OD_600_ values of ~1). Cells were transferred to 5-ml polystyrene round-bottom tubes and were analyzed by flow cytometry for single-cell detection of *yfp* expression using a BD FACSAria IIU cell sorter. A total of 100,000 events were recorded for each sample. Experiments were performed for between 3 and 10 independent biological replicates.

### Accession numbers.

All next-generation sequencing data sets described in this paper are available at EBI ArrayExpress with the accession numbers E-MTAB-5970, E-MTAB-5971, E-MTAB-6446, E-MTAB-5972, and E-MTAB-5969.
