# Supplementary figures and images for: Determining the Specificity of Cascade Binding, Interference, and Primed Adaptation In Vivo in the Escherichia coli Type I-E CRISPR-Cas System
Source: mBio. 2018 Apr 17;9(2):e02100-17. doi: 10.1128/mBio.02100-17 (PMC5904413; doi:10.1128/mBio.02100-17)

Figure S1

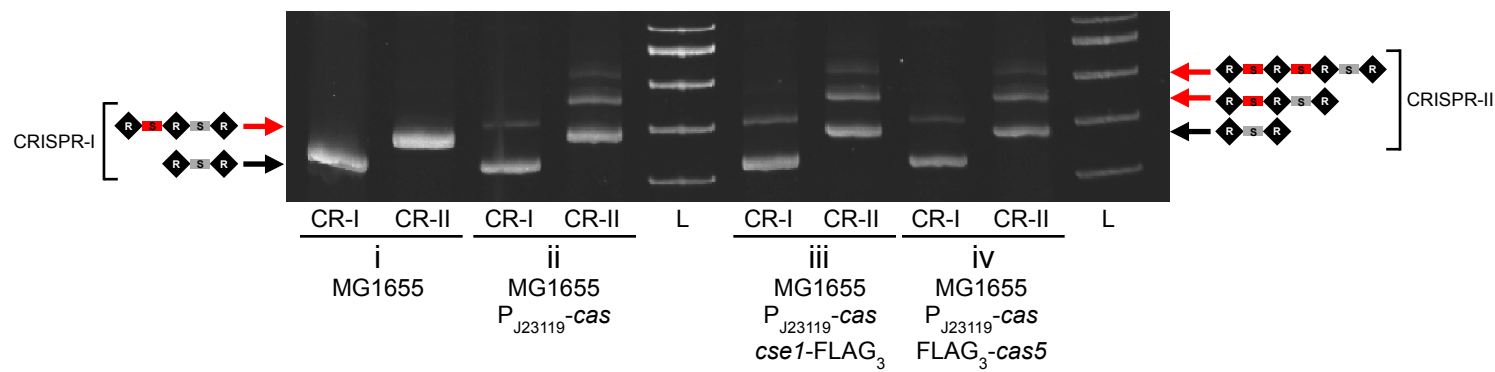

Supplement: FIG S1 [file mbo002183842sf1.pdf]

Figure S3

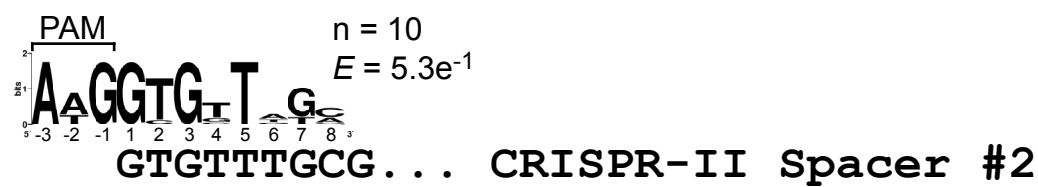

Supplement: FIG S3 [file mbo002183842sf3.pdf]

Figure S4

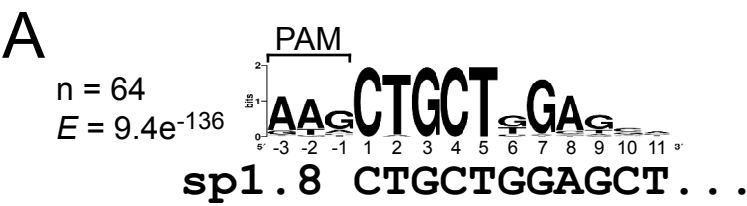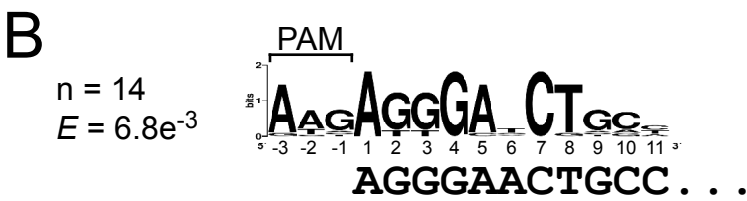

Supplement: FIG S4 [file mbo002183842sf4.pdf]
