# Supplementary material for: Determining the Specificity of Cascade Binding, Interference, and Primed Adaptation In Vivo in the Escherichia coli Type I-E CRISPR-Cas System
Source: mBio. 2018 Apr 17;9(2):e02100-17. doi: 10.1128/mBio.02100-17 (PMC5904413; doi:10.1128/mBio.02100-17)
Supplement: FIG S2 [file mbo002183842sf2.pdf]

Figure S2

A

lacZ-targeting crRNA spacer: 5′ CTTTACACTTTATGCTTCCGGCTCGTATGT 3′

B

araB-targeting crRNA spacer: 5′ ATTAGCGGATCCTACCTGACGCTTTTTATC 3′

C

CRISPR-I

GAGTTCCCCGCGCCAGCGGGGATAAACCGCTTTCGCAGACGCGCGGCGATACGCTCACGCA**GAG**
TTCCCCGCGCCAGCGGGGATAAACCGCAGCCGAAGCCAAAGGTGATGCCGAACACGCT**GAGTTC**
CCCGCGCCAGCGGGGATAAACCGGGCTCCCTGTCGGTTGTAATTGATAATGTTGA**GAGTTCCCC**
GCGCCAGCGGGGATAAACCGTTTGGATCGGGTCTGGAATTTCTGAGCGGTCGC**GAGTTCCCCGC**
GCCAGCGGGGATAAACCGCGAATCGCGCATACCCTGCGCGTCGCCGCCTGC**GAGTTCCCCGCGC**
CAGCGGGGATAAACCGTCAGCTTTATAAATCCGGAGATACGGAAACTA**GAGTTCCCCGCGCCAG**
CGGGGATAAACCGGACTCACCCCGAAAGAGATTGCCAGCCAGCTT**GAGTTCCCCGCGCCAGCGG**
GGATAAACCGCTGCTGGAGCTGGCTGCAAGGCAAGCCGCCCA**GAGTTCCCCGCGCCAGCGGGGA**
TAAACCGGGGGGCGCATGACCGTAAACATTATCCCCCGG**GAGTTCCCCGCGCCAGCGGGGATAA**
ACCGGGAGTTCAGACATAGGTGGAATGATGGACTAC**GAGTTCCCCGCGTTAGCGGGGATAAACC**
GCCCGGTAGCCAGGTTTGCAACGCCTGAACCGA**GAGTTCCCCGCGCCAGCAGGGATAAACCG**GC
AACGACGGTGAGATTTCACGCCTGACGCTG**GTGTTCCCCGCATCAGCGGGGATAAACCG**GGCGC
ACTGGATGCGATGATGGATATCACTTG**GAGTTCCCCCGCCTCTGCGGTAGAACTCC**

D

CRISPR-II

GTGTTCCCCGCGCCAGCGGGGATAAACCGGCAAAAACCGGGCAATCGCAAAAAGGCGTAAT**GTG**
TTCCCCGCGCCAGCGGGGATAAACCTGTGTTTGCGGCATTAACGCTCACCAGCATTTC**GTGTTC**
CCCGCGCCAGCGGGGATAAACCGACGTGGTCATGGGTGCTGCTGTTGCAGAGCCA**GTGTTCCCC**
GCGCCAGCGGGGATAAACCGAGCAGATACACGGCTTTGTATTCCGTGCGCCC**GTGTTCCCCGCG**
CCAGCGGGGATAAACCGAATAGCAATAGTCCATAGATTTGCGAAAACAG**GTGTTCCCCGCGCCA**
GCGGGGATAAACCGGAGCCTGACGAGACTACTGAGGCCGTTCTGTC**GAGTTCCCCGCGCCAGCG**
GGGATAAACCA

E

Portion of CRISPR-I spacer #8-expressing crRNA plasmid

GAGTTCCCCGCGCCAGCGGGGATAAACCGCTGCTGGAGCTGGCTGCAAGGCAAGCCGCCTC**GAG**
TTCCCCGCGCCAGCGGGGATAAACCGAGGGAACTGCCAGGCATCAAATAAAACGAAAGGCTCAG
TCGAAAGACTGGGCCTTTCGTTTT
