# Supplementary material for: Determining the Specificity of Cascade Binding, Interference, and Primed Adaptation In Vivo in the Escherichia coli Type I-E CRISPR-Cas System
Source: mBio. 2018 Apr 17;9(2):e02100-17. doi: 10.1128/mBio.02100-17 (PMC5904413; doi:10.1128/mBio.02100-17)
Supplement: TABLE S4 [file mbo002183842st4.pdf]

Table S4. Numbers of potential off-target chromosomal binding sites for spacers in the CRISPR-I array.

	5 bp[a]	6 bp[a]	7 bp[a]	8 bp[a]	9 bp[a]	10 bp[a]
**Spacer 1**	112	35	18	6	1	0
**Spacer 2**	157	41	7	3	1	0
**Spacer 3**	16	3	0	0	0	0
**Spacer 4**	87	7	2	0	0	0
**Spacer 5**	181	53	13	3	0	0
**Spacer 6**	183	27	13	4	2	0
**Spacer 7**	36	13	5	0	0	0
**Spacer 8**	232	133	46	14	5	0
**Spacer 9**	33	2	0	0	0	0
**Spacer 10**	57	18	2	1	1	1
**Spacer 11**	207	65	15	4	0	0
**Spacer 12**	152	54	10	1	1	1
**Spacer 13**	123	32	3	1	1	0

	7 bp[a,b]	8 bp[a,b]	9 bp[a,b]	10 bp[a,b]
**Spacer 1**	25	7	1	0
**Spacer 2**	28	8	5	1
**Spacer 3**	4	0	0	0
**Spacer 4**	23	6	3	0
**Spacer 5**	35	11	2	0
**Spacer 6**	43	11	4	1
**Spacer 7**	8	2	0	0
**Spacer 8**	74	23	7	1
**Spacer 9**	5	0	0	0
**Spacer 10**	5	2	1	1
**Spacer 11**	70	25	10	1
**Spacer 12**	46	14	7	1
**Spacer 13**	16	5	3	0

[a] Number of chromosomal loci with an AAG PAM flanked by an identical sequence match to the specific length from the start of the indicated spacer.
[b] Number of chromosomal loci with an AAG PAM flanked by an identical sequence match to the specific length from the start of the indicated spacer, allowing for a mismatch at position 6.
