# Supplementary material for: Determining the Specificity of Cascade Binding, Interference, and Primed Adaptation In Vivo in the Escherichia coli Type I-E CRISPR-Cas System
Source: mBio. 2018 Apr 17;9(2):e02100-17. doi: 10.1128/mBio.02100-17 (PMC5904413; doi:10.1128/mBio.02100-17)
Supplement: TABLE S5 [file mbo002183842st5.pdf]

Table S5. Strains, Plasmids, Oligonucleotides, and Chemically Synthesized dsDNA fragments used in this study.

Strains

**Name**	**Description**	**Source**
MG1655	MG1655 (F− λ− Δ*ilvG* *rfb*-50 *rph*-1)	(1)
CB386	MG1655 [Δ*cas3* P*cse1*]::[*cat* PJ23119]	(2)
MLS1003	MG1655 *araB*::T7pol_*tetA* Δ*araA* Δ*cas3* ΔCRISPR-I ΔCRISPR-II *galK*::J23101_L-II_RII_link_SYFP_op	(3)
AMD688	MLS1003 [Δ*cas3* P*cse1*]::[PJ23119]	This Study
AMD536	MG1655 [Δ*cas3* P*cse1*]::[PJ23119]	This Study
AMD543	CB386 Cse1-$FLAG_3$	This Study
AMD554	CB386 $FLAG_3$-Cas5	This Study
LC060	AMD536 Cse1-$FLAG_3$ ΔCRISPR-II	This Study
LC074	AMD536 ΔCRISPR-I	This Study
LC077	LC074 Cse1-$FLAG_3$	This Study
AMD566	AMD536 Cse1-$FLAG_3$	This Study
LC099	AMD566 *yggX**	This study
LC103	AMD536 Δ*yggX*::*kan*^{R}	This Study
LC106	LC103 Δ*cas1*	This Study

Plasmids

**Name**	**Description**	**Source**
pBAD24 amp	Empty pBAD24	(4)
pCB380	pcrRNA.con-*lacZ*	(2)
pCB381	pcrRNA.con-*araB*	(2)
pAMD172	Plasmid with synthesized DNA Fragment	This Study
pAMD179	Parent vector for cloning crRNAs	This Study

pLC008	pAMD179 expressing wild-type sp1.8	This Study
pLC010	pAMD179 expressing mutant sp1.8	This Study
pAMD189	pAMD179 expressing a self-targeting crRNA	This Study
pLC021	pBAD24 with protospacer matching the off-target site from *yggX* (includes AAG PAM)	This Study
pLC022	pBAD24 with protospacer that is a perfect match to sp1.8, with an AAG PAM	This Study
pBAD33 Cam	pBAD33 Cam	(4)
pAMD191	pBAD33-*cas3*	This Study
pLC020	"Pre-protospacer" Plasmid	This Study
pLC023	Derivative of pLC020 with optimal protospacer matching sp1.8 (variant i)	This Study
pLC024	Derivative of pLC020 with protospacer matching sp1.8 with mismatches across positions 25-32 (variant ix)	This Study
pLC025	Derivative of pLC020 with protospacer matching sp1.8 with mismatches across positions 19-32 (variant x)	This Study
pLC026	Derivative of pLC020 with protospacer matching sp1.8 with mismatches across positions 1-6 (variant xi)	This Study
pLC027	Derivative of pLC020 with protospacer matching sp1.8 with a CCG PAM (variant ii)	This Study
pLC028	Derivative of pLC020 with protospacer with mismatches across positions 1-6 and 25-32 (variant xii)	This Study
pLC029	Derivative of pLC020 with protospacer matching sp1.8 with an ATT PAM (variant iii)	This Study
pLC030	Derivative of pLC020 with protospacer with mismatches across positions 7-24 (variant xiii)	This Study
pLC031	Derivative of pLC020 with protospacer matching sp1.8 with seed mismatches (GGT; variant iv)	This Study

pLC032	Derivative of pLC020 with protospacer matching sp1.8 with seed mismatches (CGC; variant vii)	This Study
pLC033	Derivative of pLC020 with protospacer matching sp1.8 with seed mismatches (GTC; variant v)	This Study
pLC034	Derivative of pLC020 with protospacer matching sp1.8 with seed mismatches (CCT; variant viii)	This Study
pLC035	Derivative of pLC020 with protospacer matching sp1.8 with seed mismatches (TTT; variant vi)	This Study
pCP20	pPC20	(5)
pLC057	pBAD24 with protospacer that is a perfect match to CRISPR-I sp1.2, with an AAG PAM	This Study

Oligonucleotides and Chemically Synthesized dsDNA fragments

**Name**	**Sequence**
JW6272	GTTAAAATCCTGGGTTCGTAATAATGGCGAGGCGTGAACATGAGAGGCGGTGGCGACTAC
JW6273	CCCCCAGGCTTGCATTGGCCCAGCAAGCCGCAAGATCAAATAAGACGCCGCCTTGTCATC
JW6364	GCGCTTGCCCGCGCCACGCTATACAAACATTTACGGGAGTTAAAGGCGGTGGCGACTAC
JW6365	GCATCAATTTCATCAGCCATTTGATGGCCCTCCTTGCGGGTTGGGAGCTCACTACTTGTC
JW6421	GTTTTTTTGGGCTAGCGAGTTC
JW6513	CCCGTTTTTTTGGGCTAGGGAGTTC
JW6518	CAGCGGGGATAAACC
JW7490	AAACCATGCTGATTAATGAAA
JW7491	TCGATATGCACCTCTTTACC

JW7529	TTTATGGGAAAAAATGCTTTAAGAACAAATGTATACTTTTAGATAGACAGCTGCATGCAT
JW7530	GCGGGGAACACCAGCGTCAGGCGTGAAATCTCACCGTCGTTGCGTGTAGGCTGGAGCTG
JW7537	ACTGGCTTAAAAAATCATTAATTAATAATAGGTTATGTTTAGATAGACAGCTGCATGCAT
JW7538	TGCTAATATAAAAACTTGAGAAAGAGATAACGGGTTATATGGTGTGTAGGCTGGAGCTG
JW7539	ATCATTAATTAATAATAGGTTATGTTTAGAACCATATAACCCGTTATCTCTTTCTCAAGT
JW7540	ACTTGAGAAAGAGATAACGGGTTATATGGTTCTAAACATAACCTATTATTAATTAATGAT
JW7598	GTTTTTTTGGGCTAGGGAGTTCCCCGCGCCAGCGGGGATAAACCG
JW7635	GATTAATGAAAAGAAACTCAACATGATGAATGCCGAGCACCGCTAGACAGCTGCATGCAT
JW7636	ATCTTCCGGCGTATAGCCCTCGATATGCACCTCTTTACCCTCGGTGTAGGCTGGAGCTG
JW7637	GATGAATGCCGAGCACCGCAAGTGACTTGAGCAGGAGATGGTCAAC
JW7638	GTTGACCATCTCCTGCTCAAGTCACTTGCGGTGCTCGGCATTCATC
JW7693	GCGCGGGGAACTCGACTGGTGAGTACTCAACCAAGTCATTCTGAGCGGTTTATCCCCGC
JW7736	GGGCTAGCGAATTCGAAAAACAGGGAGGCTATTAAT
JW7738	GATCCCCGGGTACCGTTATTTGGGATTTGCAGGGA
JW7818	TCGTCGGCAGCGTCAGATGTGTATAAGAGACAGAAAGTTGGTAGATTGTGACTGGC
JW7819	GTCTCGTGGGCTCGGAGATGTGTATAAGAGACAGCAACAGCAGCACCCATGAC
JW7898	TGGCTTGCTATCTTTGGCTCCACTGTGATTGAGGTGTAATAAATAGACAGCTGCATGCAT
JW7899	GAGGTACATTTTCAGTGACCACGACCAACATACTCATTGTGTAGGCTGGAGCTG
JW7900	TCCACTGTGATTGAGGTGTAATAAAAATGAGTATGTTGGTCGTGGTCG
JW7901	CGACCACGACCAACATACTCATTTTTATTACACCTCAATCACAGTGGA
JW7911	GCGCGGGGAACTCGAGGCGGCTTGCCTTGCAGCCAGCTCCAGCAGCGGTTTATCCCCGC
JW7912	GCGCGGGGAACTCGAGGCGGCTTGCCTTGCAGCCAGCTCCAGTCACGGTTTATCCCCGC
JW7938	TGCGACGCTGGCGATATCTGAATGCCGAGCACCGCA
JW7939	AAACAGCCAAGCTTGCATGCTGCACCTCTTTACCCTCG
JW7922	ATGCACGAAATAACCCTCT
JW7923	TCACATACTGTTGGCATGTT
JW7940	TGCGACGCTGGCGATATCCGAGCACCGCAAGCTGCTGGAGCTGGCTGCAAGGCAAGCCGC
JW7941	AAACAGCCAAGCTTGCATGTGCACCTCTTTACCCTCGTGGGCGGCTTGCCTTGCAGCCAG

JW8040	CTAGCAGGAGGAATTCTGATTTTATCGCACCACTC
JW8042	CGTCGGTTTTTTTACCCTC
JW8043	GTACCATGGTGAATTTCGCTCATTTCGACTTCGG
JW8128	GTAAAAAAACCGACGGAATTCGAAACGTGTTGCTGTGGG
JW8129	CGTTTCCTGAGAATTNNNNNGTCTCGACTGAGGAACCATGAAACAGTATTTAGAACTG
JW8130	AAAACCGACGGAATTGAAGCTGCTGGAGCTGGCTGCAAGGCAAGCCGCCCAACACACTGGCGTCGGCTC T
JW8139	AAAACCGACGGAATTGAAGCTGCTGGAGCTGGCTGCAAGGCAAAGTATATGCCACACTGGCGTCGGCTC T
JW8145	AAAACCGACGGAATTGAAGCTGCTGGAGCTGGCTGCAGACTTCAGTATATGCCACACTGGCGTCGGCTC T
JW8169	AAAACCGACGGAATTGAAGTGATGCGAGCTGGCTGCAAGGCAAGCCGCCCAACACACTGGCGTCGGCTC T
JW8499	AAAACCGACGGAATTGCCGCTGCTGGAGCTGGCTGCAAGGCAAGCCGCCCAACACACTGGCGTCGGCTC T
JW8500	AAAACCGACGGAATTGAAGTGATGCGAGCTGGCTGCAAGGCAAAGTATATGACACACTGGCGTCGGCTC T
JW8501	AAAACCGACGGAATTGATTCTGCTGGAGCTGGCTGCAAGGCAAGCCGCCCAACACACTGGCGTCGGCTC T
JW8502	AAAACCGACGGAATTGAAGCTGCTGACTGATATCATTGACTTCGCCGCCCAACACACTGGCGTCGGCTCT
JW8537	CAAGCAGAAGACGGCATACGAGATGGATTCACGTCTCGTGGGCTCGGAGATGTGTATAAGAGACAGCGG AGGGTAAAAAAACCGACGGAATT
JW8556	CAAGCAGAAGACGGCATACGAGATGCTAGACTGTCTCGTGGGCTCGGAGATGTGTATAAGAGACAGCGG AGGGTAAAAAAACCGACGGAATT
JW8557	CAAGCAGAAGACGGCATACGAGATCTCAGGTAGTCTCGTGGGCTCGGAGATGTGTATAAGAGACAGCGG AGGGTAAAAAAACCGACGGAATT
JW8558	CAAGCAGAAGACGGCATACGAGATCGCATTAGGTCTCGTGGGCTCGGAGATGTGTATAAGAGACAGCGG AGGGTAAAAAAACCGACGGAATT
JW8559	CAAGCAGAAGACGGCATACGAGATTGTCCAAGGTCTCGTGGGCTCGGAGATGTGTATAAGAGACAGCGG AGGGTAAAAAAACCGACGGAATT
JW8561	CAAGCAGAAGACGGCATACGAGATGGCTATCAGTCTCGTGGGCTCGGAGATGTGTATAAGAGACAGCGG AGGGTAAAAAAACCGACGGAATT

JW8562	CAAGCAGAAGACGGCATACGAGATAACTCGTGGTCTCGTGGGCTCGGAGATGTGTATAAGAGACAGCGG<br>AGGGTAAAAAAACCGACGGAATT
JW8563	CAAGCAGAAGACGGCATACGAGATATGGACTCGTCTCGTGGGCTCGGAGATGTGTATAAGAGACAGCGG<br>AGGGTAAAAAAACCGACGGAATT
JW8564	CAAGCAGAAGACGGCATACGAGATCATATGGCGTCTCGTGGGCTCGGAGATGTGTATAAGAGACAGCGG<br>AGGGTAAAAAAACCGACGGAATT
JW8565	CAAGCAGAAGACGGCATACGAGATTAAGGCGAGTCTCGTGGGCTCGGAGATGTGTATAAGAGACAGCG<br>GAGGGTAAAAAAACCGACGGAATT
JW8566	CAAGCAGAAGACGGCATACGAGATCGTACTAGGTCTCGTGGGCTCGGAGATGTGTATAAGAGACAGCGG<br>AGGGTAAAAAAACCGACGGAATT
JW8567	AATGATACGGCGACCACCGAGATCTACACTCCAGGTATCGTCGGCAGCGTCAGATGTGTATAAGAGACA<br>GGACTTTGAGATTGAAGGCTACGATCCG
JW8675	AAAACCGACGGAATTGAAGGGTCTGGAGCTGGCTGCAAGGCAAGCCGCCCAACACACTGGCGTCGGCTC<br>T
JW8676	AAAACCGACGGAATTGAAGCGCCTGGAGCTGGCTGCAAGGCAAGCCGCCCAACACACTGGCGTCGGCTC<br>T
JW8677	AAAACCGACGGAATTGAAGGTCCTGGAGCTGGCTGCAAGGCAAGCCGCCCAACACACTGGCGTCGGCTC<br>T
JW8678	AAAACCGACGGAATTGAAGCCTCTGGAGCTGGCTGCAAGGCAAGCCGCCCAACACACTGGCGTCGGCTC<br>T
JW8679	AAAACCGACGGAATTGAAGTTTCTGGAGCTGGCTGCAAGGCAAGCCGCCCAACACACTGGCGTCGGCTC<br>T
144148263 (synthesized dsDNA fragment)	CAGACATTTGGGTTAAACAGGCGTACCCCGGTAGATTTGGATGGTTTAAGGTTGGTGTCTTTTTTACCTGT<br>TTGAAAACAAAGAATTAGCTGATCTTTAATAATAAGGAAATGTTACATTAAGGTTGGTGGGTTGTTTTTA<br>TGGGAAAAAATGCTTTAAGAACAAATGTATACTTTTAGAGAGTTCCCCGCGCCAGCGGGGATAAACCGC<br>AGCTCCCATTTTCAAACCCATCAAGACGCCTTCGCCAACTCCTTCACCAGAGGTAGCATTATCCGCATAA<br>CGTCACGGCAGCGACGTTCTATTCTTCCAGGAAGAGCCTTATCAATATGTTGGTGATTATCCAGTCTTAC<br>GTCATGCCAGCTATTTCCCGCCGGGAAGGCAGGTGTTTTTGCGCG
Chemically synthesized insert from pAMD172	TCGTAATACGACTC_CTATAGACAACGGTCAGGAGAAGGAGGACGGCATGTACCCATACGACGTCCCAG<br>ACTACGCTAGTACTGACTACAAGGATCACGACTACAAGGACCACGACTATAAAGACCACGACGCAATGT<br>AGGTTTTTTTGGGCTAGCGAGTTCCCCGCGCCAGCGGGGATAAACCGCGGAATAATAATAATCTCGAGTT<br>CCCCGCGCCAGCGGGGATAAACCGAGGGAACTGCCAGGCATCAAATAAAACGAAAGGCTCAGTCGAAA<br>GACTGGGCCTTTCGTTTTATCTGTTGTTTGTCGGTGAACGCTCTCCTGAGTAGGACAAGCTTGGCTGTTTT

	GGCATAATACGACTCACTATAGTGAACGGTCTCCCCGTCCTC_ACGGCGACTACCCATACGACGTCCCAGACTACGCTAGTACTGACTACAAGGATCACGACTACAAGGACCACGACTATAAAGACCACGACGCAATGTA_TCACACTGG
JW9131	TGCGACGCTGGCGATATCCGAGCACCGCAAGCAGCCGAAGCCAAAGGTGATGCCGAACAC
JW9132	AAACAGCCAAGCTTGCATGTGCACCTCTTTACCCTCGAGCGTGTTCGGCATCACCTTTG

REFERENCES

1. Blattner FR, Plunkett G, Bloch CA, Perna NT, Burland V, Riley M, Collado-Vides J, Glasner JD, Rode CK, Mayhew GF, Gregor J, Davis NW, Kirkpatrick HA, Goeden MA, Rose DJ, Mau B, Shao Y. 1997. The complete genome sequence of Escherichia coli K-12. Science 277:1453–1462.

2. Luo ML, Mullis AS, Leenay RT, Beisel CL. 2014. Repurposing endogenous type I CRISPR-Cas systems for programmable gene repression. Nucleic Acids Res gku971.

3. Amlinger L, Hoekzema M, Wagner EGH, Koskiniemi S, Lundgren M. 2017. Fluorescent CRISPR Adaptation Reporter for rapid quantification of spacer acquisition. Sci Rep 7:10392.

4. Guzman LM, Belin D, Carson MJ, Beckwith J. 1995. Tight regulation, modulation, and high-level expression by vectors containing the arabinose PBAD promoter. J Bacteriol 177:4121–4130.

5. Cherepanov PP, Wackernagel W. 1995. Gene disruption in Escherichia coli: TcR and KmR cassettes with the option of Flp-catalyzed excision of the antibiotic-resistance determinant. Gene 158:9–14.
